# Gender Differences in Primary Care Physician Earnings and Outcomes Under Medicare Advantage Value-Based Payment

**DOI:** 10.1001/jamahealthforum.2025.2001

**Published:** 2025-05-16

**Authors:** Ishani Ganguli, Nicholas E. Daley, Lauren Polt, Victoria DiGennaro, Benjamin Kornitzer

**Affiliations:** 1 Division of General Internal Medicine and Primary Care, Brigham and Women’s Hospital, Harvard Medical School, Boston, Massachusetts; 2 Millennium Physician Group, Fort Myers, Florida; 3 Pioneer Physicians Network, Akron, Ohio; 4 Division of General Internal Medicine, Department of Medicine, Icahn School of Medicine at Mount Sinai, New York, New York

## Abstract

This cohort study assesses gender differences in reimbursed earnings and quality among primary care physicians participating in full risk-sharing arrangements with Medicare Advantage plans, which now enroll most Medicare-eligible adults.

## Introduction

Despite some evidence of better patient outcomes, women physicians experience a large, persistent wage gap compared to their men counterparts.^1^ This gap may be due in part to still-dominant volume-based payment models: on average, women primary care physicians (PCPs) spend more time on patient care per visit and between visits, resulting in fewer billable visits and lower fee-for-service revenue.^2,3^ In theory, value-based payment models, which incentivize care quality over visit volume via flexible population-based payments and quality ratings, may improve pay equity. Specifically, a growing number of PCPs have full risk-sharing arrangements with Medicare Advantage (MA) plans in which they receive risk-adjusted, per-member-per-month payments to cover all of their patients’ medical spending. A microsimulation study showed that the gender gap may vary by type of risk adjustment used in such models,^4^ but to our knowledge, no study has examined gender differences among physicians working under value-based payment. To inform payment policy, we assessed gender differences in reimbursed earnings and quality among PCPs participating in full risk-sharing arrangements with MA plans, which now enroll most Medicare-eligible adults.

## Methods

This cross-sectional study used 2022 patient-level MA claims and Medicare Star quality data from 13 payers. We included PCPs who assumed full financial risk for their MA patients via partnership with agilon health, had documented gender, and had 50 or more MA members in their panels. The patient cohort included all MA members in these panels.

Primary outcomes were physician gender differences in per-patient earnings estimated as fee-for-service payment (reimbursement for all primary care services) and as value-based payment (earnings or losses based on their patients’ projected vs actual total medical spending), prorated to annual amounts and winsorized at 1% and 99%. Secondary outcomes included Medicare Star quality measures, utilization, and provider rating scores. Covariates included physician and patient panel characteristics (eMethods in Supplement 1).

We built multivariable logistic, linear, negative binomial, and gamma log-link regression models, adjusting for covariates and practice group fixed effects. For member-level models, we weighted or offset by member-month and clustered standard errors by physician. Mass General Brigham’s institutional review board waived review, and we followed STROBE guidelines.

## Results

We included 872 PCPs (352 women [40.4%]) working in 15 practice groups in 7 states and 223 810 patients. Men and women PCPs had similar degrees and specialties (Table 1). Women PCPs’ panels had fewer MA enrollees and higher proportions of female patients. Comparing men and women PCPs in the same practice groups, women PCPs’ patients had better hemoglobin A_1c_ control, eye examination receipt, and composite quality scores, as well as fewer emergency department visits and hospitalizations, but women received worse provider rating scores (Table 2). Women PCPs had similar earnings to men via fee for service and more earnings via value-based payment.

**Table 1.** Physician and Patient Panel Characteristics Among Men and Women Primary Care Physicians (PCPs)

Characteristic	Men PCPs (n = 520)	Women PCPs (n = 352)
**PCPs**		
Years since medical school graduation, mean (SD)	27.5 (12.0)	23.6 (9.9)
Doctor of Medicine degree, No. (%)^a^	416 (80.0)	285 (81.0)
Specialty, No. (%)		
Family medicine	326 (62.7)	224 (63.6)
Internal medicine	194 (37.3)	128 (36.4)
Practice in medically underserved area, No. (%)^b^	105 (20.2)	55 (15.6)
**Patient panels** ^c^		
MA enrollees per panel, mean (SD)	356.9 (150.2)	285.5 (161.4)
Age, mean (SD), y	75.8 (2.0)	75.4 (2.1)
Sex, mean (SD)		
% Female	48.4 (7.7)	72.9 (9.4)
% Male	51.6 (7.7)	27.0 (9.4)
CMS risk adjustment factor, mean (SD)	1.1 (0.17)	1.0 (0.17)
% With disability, mean (SD)^d^	0.35 (0.46)	0.37 (0.54)
% Eligible for Medicaid, mean (SD)	8.1 (7.0)	7.9 (6.4)

Abbreviations: CMS, Centers for Medicare & Medicaid Services; MA, Medicare Advantage.

^a^ Compared to Doctor of Osteopathic Medicine degree.

^b^ Medically underserved areas are geographic areas with a lack of access to primary care services, as defined by the Health Resources and Services Administration. Data were unknown for 19 physicians (1.9%).

^c^ For each patient panel characteristic, values were averaged across a given physician’s panel and weighted by member-months, then the mean of means for all men PCPs and for all women PCPs were estimated.

^d^ Disability status noted as reason for Medicare eligibility.

**Table 2.** Quality, Utilization, and Earnings by Primary Care Physician (PCP) Gender^a^

Outcome	Total No.	Unadjusted		Difference (95% CI)^b^	
		Men PCPs (n = 520)	Women PCPs (n = 352)	Unadjusted	Adjusted^c^
**Quality**					
Hypertension medication adherence among eligible patients, No. (%)^d^	61 421	37 267 (92.0)	19 149 (92.3)	0.29 (−0.30 to 0.88)	0.17 (−0.43 to 0.77)
Diabetes medication adherence among eligible patients, No. (%)^d^	20 993	12 463 (89.7)	6330 (89.5)	−0.21 (−1.3 to 0.84)	−0.15 (−1.2 to 0.88)
Hemoglobin A_1c_ control among eligible patients, No. (%)^d^	23 658	14 436 (92.2)	7536 (94.2)	2.0 (1.0 to 2.9)	1.3 (0.48 to 2.2)
Eye examination receipt among eligible patients, No. (%)^d^	23 656	12 164 (77.8)	6475 (81.3)	3.5 (2.0 to 5.0)	2.8 (1.3 to 4.3)
Quality composite Stars measure among eligible patients, No. (%)^d^	152 824	92 951 (92.6)	48 495 (93.2)	0.61 (0.15 to 1.1)	0.57 (0.16 to 0.97)
Patient-reported physician rating among eligible physicians, mean (SD)^e^	478	87.8 (6.9)	87.7 (7.3)	−0.01 (−1.3 to 1.3)	−2.7 (−5.0 to −0.45)
**Utilization** ^f^					
Primary care visits per patient, mean (SD)	223 810	3.9 (4.5)	3.8 (4.4)	−2.6 (−6.5 to 1.3)	−2.8 (−5.9 to 0.37)
ED visits per 1000 patients, mean (SD)	223 810	693.1 (1866.3)	651.5 (1799.6)	−6.4 (−12.2 to −0.56)	−5.5 (−9.1 to −1.7)
Hospitalizations per 1000 patients, mean (SD)	223 810	182.9 (649.9)	153.9 (596.8)	−15.9 (−20.9 to −11.0)	−7.7 (−11.4 to −3.8)
**Payment**					
Fee-for-service earnings per patient, mean (SD), $ per year^g^	185 922	475.86 (438.15)	454.49 (413.39)	−21.37 (−44.25 to 1.52)	−13.08 (−29.51 to 3.34)
Value-based payment earnings per patient, mean (SD), $ per year^h^	185 922	1931.78 (17 176.69)	2236.45 (15 934.93)	304.67 (−4.43 to 613.77)	275.11 (57.97 to 492.25)

Abbreviation: ED, emergency department.

^a^ *P* < .05 was considered statistically significant, and Snowflake, version 8, and R, version 4.3.2 (R Project for Statistical Computing), were used for analyses.

^b^ The difference indicates the estimate for women PCPs minus the estimate for men PCPs; differences in quality outcomes (other than patient-reported physician rating) are reported in percentage points; differences in utilization are reported as percentages.

^c^ Adjusted models included physician time since medical school graduation, degree type, specialty, practice site in a medically underserved area, and patient age, sex, Centers for Medicare & Medicaid Services Risk Adjustment factor, disability status, and Medicaid eligibility, as well as practice group fixed effects. Outputs were estimated as adjusted marginal differences. Missingness was addressed using the indicator variable method.

^d^ For binary member-level quality outcomes, logistic regression models weighted by member-month (as per Centers for Medicare & Medicaid Services protocol) were run, and standard errors were clustered by physician.

^e^ For physician-level patient-reported provider rating scores, linear regression models, both unadjusted and adjusted for physician characteristics and patient panel characteristics, were run. NRC Health surveys patients using the validated CAHPS (Consumer Assessment of Healthcare Providers and Systems) survey. These data were available for a subset of all study physicians (295 men and 183 women).

^f^ For utilization outcomes, unadjusted rates by months enrolled in Medicare Advantage were prorated, and results were winsorized at 99%. For utilization models, negative binomial regressions offset by member-month were run, and standard errors were clustered by physician.

^g^ Fee-for-service earnings were prorated to annual amounts and winsorized at 1% and 99%; gamma log-link models weighted by member-month were run, and standard errors were clustered by physician. Estimated among patients with payers that provided complete payment data.

^h^ Value-based payment earnings (which can be positive or negative) were prorated to annual amounts and winsorized at 99%; ordinary least-squares regression models weighted by member-month were run, and standard errors were clustered by physician. Estimated among patients with payers that provided complete payment data.

## Discussion

In this cohort study, women PCPs in value-based payment models had equal or better quality outcomes and higher value-based earnings compared to men in their practice groups. These results substantiate prior evidence that women physicians perform better on process and outcome measures, yet receive incommensurate patient ratings.^5,6^ The reversal of the gender gap under value-based payment is likely due to fewer emergency department visits and hospitalizations among women PCPs’ patients and may in turn reflect better alignment of value-based models to practice patterns more common in women (eg, more face-to-face time per visit).^2^ Equal pay could carry benefits beyond fair compensation, including reduced burnout and improved retention of the increasingly female primary care workforce to care for the aging US population.

Limitations include that results may not generalize beyond physicians choosing full-risk arrangements, MA enrollees represent a portion of panels, and we cannot observe how practice groups translated payments to take-home wages (though comparing PCPs within practice groups mitigates this concern). These results support the possibility that growing use of value-based payment might improve the gender wage gap and better reward high-quality care. Future studies should explore how outcomes change with greater shares of patients in these arrangements.

## Notes

Supplement 1.
eMethods

eReferences

Supplement 2.
Data Sharing Statement
