# Errors in Table 1

**DOI:** 10.1001/jamahealthforum.2025.2715

**Published:** 2025-06-27

The Research Letter titled “Gender Differences in Primary Care Physician Earnings and Outcomes Under Medicare Advantage Value-Based Payment,”^1^ which published May 16, 2025, contained data errors in Table 1. The correct No. (%) of men and women primary care physicians who practice in medically underserved areas are 105 (20.2%) and 55 (15.6%), respectively. This article has been corrected.
