# Supplementary material for: Gender Differences in Primary Care Physician Earnings and Outcomes Under Medicare Advantage Value-Based Payment
Source: JAMA Health Forum. 2025 May 16;6(5):e252001. doi: 10.1001/jamahealthforum.2025.2001 (PMC12084836; doi:10.1001/jamahealthforum.2025.2001)
Supplement: Supplement 1. — eMethods eReferences [file jamahealthforum-e252001-s001.pdf]

Supplemental Online Content

Ganguli I, Daley NE, Polt L, DiGennaro V, Kornitzer B. Gender differences in primary care physician earnings and outcomes under Medicare Advantage value-based payment. *JAMA Health Forum*. 2025;6(5):e252001. doi:10.1001/jamahealthforum.2025.2001

eMethods

eReferences

This supplemental material has been provided by the authors to give readers additional information about their work.

© 2025 Ganguli I et al. *JAMA Health Forum*.

eMethods

Description of Medicare Advantage and Full Risk-Sharing Arrangements – Medicare Advantage (MA) is an insurance program in which the federal Centers for Medicare and Medicaid Services (CMS) contracts with private payers to administer insurance for Medicare-eligible beneficiaries. MA is an alternative to Traditional (Fee-For-Service) Medicare, which CMS administers directly. As of 2024, 54% of all eligible Medicare beneficiaries (i.e., 32.8 million out of 61.2 million total Medicare beneficiaries) are in MA plans.[1]

CMS negotiates risk contracts with MA plans. Under these contracts, CMS pays the MA plans pre-set, risk-adjusted per-member-per-month payments for each MA member (using historical spending benchmarks that are adjusted by the Medicare Risk Adjustment Factor, which accounts for demographics and medical complexity, as well as by potential quality bonus payments (see below re: Quality measures)). In return, the MA plans agree to assume the full risk of covering their members' total medical expenditures. MA plans can then share this risk with physician groups to varying extents. This includes the increasingly common approach[2] of primary care physician (PCP) groups taking on full risk-sharing arrangements, often with the assistance of care-enablement companies like agilon health ("agilon").

PCP groups in these full risk-sharing arrangements are guaranteed the pre-set, risk-adjusted per-member-per-month payments from MA plans for their MA-enrolled patients, minus a pre-negotiated percentage that the plans keep. In turn, the PCP groups are accountable for total medical expenditures (not limited to primary care spending) incurred by these patients. Put another way, the PCPs are at full upside and downside risk for their patients' total medical spending (Alternative Payment Model Category 4[3]), meaning they are responsible for paying for any services that exceed the set amounts they receive, and, if expenditures are below that amount, they share in the savings. This is generally operationalized by the plans paying PCPs fee-for-service reimbursements for all primary care services they provide, then reconciling their MA patients' total medical spending against the pre-set payments at the end of a given year to determine PCPs' earnings or losses under value-based payment.

These full risk-sharing arrangements are designed to give PCPs the flexibility to use innovative approaches to improve care quality and clinical outcomes rather than focusing on maximizing visit and procedure volume to generate earnings. Because revenue is pre-determined for the patient population at risk, practices can proactively invest in staff members and care resources, such as nurses, social workers, and technology, to improve the quality and efficiency of care.

Description of practices – The primary care practices included in this study were partnered with agilon, a public, value-based care-enablement company that operates in 13 states: Ohio, Texas, Connecticut, North Carolina, Pennsylvania, Maine, South Carolina, Minnesota, Michigan, Tennessee, Georgia, Kentucky, and New York. Practices in this study operated in 7 states: Ohio, Texas, Connecticut, North Carolina, Pennsylvania, Michigan, and New York. agilon shares risk with the practices and provides resources to support value-based care transformation, including training, technology, data insights, disease-specific clinical programs, and payer contracting.

Patient attribution – Patients with available payer claims data were attributed to PCPs based on their 2022 designation in each practice's electronic medical record or practice management program during the study period.

© 2025 Ganguli I et al. *JAMA Health Forum*.

***Quality measures* –** MA plans are required to report the quality of care received by their members using the Star Ratings system developed by CMS. Plans with high performance on these metrics can receive a 5% quality bonus from CMS, which is reflected in per-member-per-month payments.[4] In this study, we evaluated four Star quality measures that apply to primary care as well as the Star Composite measure to examine gender differences in patient outcomes (see Supplement Table). We did this to contextualize the implications of any differences in earnings between the groups (e.g., to understand if any differences may come at the expense of care quality).

© 2025 Ganguli I et al. *JAMA Health Forum*.

Measure Definitions and Sources

Measure	Definition	Source
**Physician Characteristics**		
Physician years since medical school graduation	Total number of years since physician graduated from medical school	Medicare Care Compare National Downloadable File linked by National Provider Identifier (NPI) https://data.cms.gov/provider-data/dataset/mj5m-pzi6
Degree type	Medical Doctor (MD), Doctor of Osteopathy (DO)	agilon provider roster
Specialty	Family medicine, internal medicine, other (Infectious Disease, Nephrology, and Hematology & Oncology)	agilon provider roster
Practice in Medically Underserved Area	Yes or No. A Medically Underserved Area is defined by the Health Resources and Services Administration as a geographic area with lack of access to primary care services. Linked by zip code to the provider practice location.	https://bhw.hrsa.gov/workforce-shortage-areas/shortage-designation#mups
**Patient Characteristics**		
Age	Whole number, in years	MA payers member attribution file
Sex	Male, Female	MA payers member attribution file
Risk Adjustment Factor	Risk Adjustment Factor (RAF), which is used to estimate the cost to treat a patient in a given year based on the patient's specific health needs. Each enrollee risk score is based on the individual's demographic and health status information. A risk score is calculated as the sum of these demographic and health factors weighted by their estimated marginal contributions to total risk.	https://www.cms.gov/CCIIO/Resources/Presentations/Downloads/hie-risk-adjustment-methodology.pdf
Disability	Yes or No, as reason for Medicare eligibility. Yes = patient has at least one disability, per CMS requirements.	https://www.cms.gov/files/document/2015-medicare-other-programs-people-disabilities-workbookpdf
Medicaid eligibility	Yes or No; Yes = eligible for Medicaid, per CMS eligibility criteria.	https://www.medicaid.gov/medicaid/eligibility/index.html
**Quality**		
Medication adherence for hypertension (RAS antagonists)	Among MA enrollees aged 18 - 85 years with a diagnosis of hypertension and a prescription for a RAS Antagonist (defined as at least 2 medication fills on unique dates of service), the percentage with Proportion of Days Covered of 80% or higher during the measurement period.<br>Star rating component.	https://www.cms.gov/files/document/2024technotes20230929.pdf
Medication adherence for diabetes medications	Among MA enrollees 18 years of age and older with a prescription for diabetes medication (defined as at least 2 medication fills, on unique dates of service, of biguanides, sulfonylureas, thiazolidinediones, DPP-4 Inhibitors, GLP-1 agonists, meglitinides, or SGLT2 inhibitors), the percentage with Proportion of Days Covered of 80% or higher during the measurement period.<br>Star rating component.	https://www.cms.gov/files/document/2024technotes20230929.pdf
Diabetes care – blood sugar controlled	Among MA enrollees aged 18 - 75 years with diabetes (type 1 and type 2), the percentage whose most recent Hemoglobin A1c level is <9% or not tested during measurement year.<br>Star rating component.	https://www.cms.gov/files/document/2024technotes20230929.pdf

© 2025 Ganguli I et al. *JAMA Health Forum*.

Diabetes care – eye exam	Among MA enrollees aged 18-75 with diabetes (type 1 and type 2), the percentage who had a retinal eye exam performed during the measurement year.<br>Star rating component.	https://www.cms.gov/files/document/2024technotes20230929.pdf
Star Rating Quality Composite Measure	Identifies proportion of eligible enrollees or events meeting requirements for Stars gaps measures, including: Breast Cancer Screening, Colorectal Cancer Screening, Controlling Blood Pressure, Follow-Up After Emergency Department Visit for People with High-Risk major complication or comorbidity, Functional Assessment, Medication Review, Medication Adherence for Diabetes Medications, Medication Adherence for Hypertension, Medication Reconciliation Post-Discharge, Medication Therapy Management, Osteoporosis Management, Pain Assessment, Statin Therapy for Patients with Cardiovascular Disease, Statin Use in Persons with Diabetes, Transition of care - Patient Engagement After Inpatient Use	https://www.cms.gov/files/document/2024technotes20230929.pdf
Patient-reported provider rating	Patient-reported overall rating of provider, administered via survey. Based on the validated Consumer Assessment of Healthcare Providers and Systems (CAHPS) Survey. Proportion of respondents who are promoters, answering 9 or 10 to this question: 'Using any number from 0 to 10, where 0 is the worst personal doctor possible and 10 is the best personal doctor possible, what number would you use to rate your personal doctor?'	NRC Health<br>https://www.ma-pdpcahps.org/globalassets/ma-pdp/current-data-collection-materials/2024/english/2024-english-ma-only-mail-survey.pdf
**Utilization**		
Primary care visits	Total number of primary care visits per patient includes in-person and virtual visits with any clinician including advanced practice clinicians, identified by the following codes: 99201-5, 99211-5, 99324-8, 99334-7, 99341-50, 99381-7, 99391-7, 99421-3, 99495-6, G0402, G0438-9, G0466-8, G2061-3	MA payer professional claim line files
Emergency department visits	The number of emergency department encounters/member months*12000, corresponding to the sum of encounters for each patient associated with emergency department utilization, normalized per 1000-member values.	MA payer institutional claim line files
Inpatient hospitalizations	The number of inpatient admits/Member months*12000, corresponding to the sum of encounters for each patient associated with inpatient hospital utilization, normalized per 1000-member values.	MA payer institutional claim line files
**Revenue**		
Fee-for-service payment	We calculated fee-for-service payment as the amount paid by insurance for services rendered by the primary care physician for each patient in the study year (2022).	MA payer claim files, total paid amounts
Value-based payment	We calculated value-based payment for each patient by adding up the patient's adjusted per-member-per-month payments for 2022, minus the pre-negotiated percent of premium (which the MA plan takes) and the patient's total medical spending in that year.	MA payer Monthly Member Report

© 2025 Ganguli I et al. *JAMA Health Forum*.

eReferences

1. Freed M, Biniek JF, Damico A, Published TN. Medicare Advantage in 2024: Enrollment Update and Key Trends. KFF. August 8, 2024. Accessed August 12, 2024. https://www.kff.org/medicare/issue-brief/medicare-advantage-in-2024-enrollment-update-and-key-trends/

2. Humana Value-Based Care Report. 2021. https://humana.gcs-web.com/static-files/39bb066e-bca5-45ff-aab2-827fe03c92c1

3. McCarron D. *Alternative Payment Model (APM) Framework*. The MITRE Corporation; 2017.

4. Biniek JF, Damico A, Published TN. Spending on Medicare Advantage Quality Bonus Payments Will Reach at Least $12.8 Billion in 2023. KFF. August 9, 2023. Accessed August 12, 2024. https://www.kff.org/medicare/issue-brief/spending-on-medicare-advantage-quality-bonus-payments-will-reach-at-least-12-8-billion-in-2023/

© 2025 Ganguli I et al. *JAMA Health Forum*.
