# Supplementary material for: Gender Differences in Primary Care Physician Earnings and Outcomes Under Medicare Advantage Value-Based Payment
Source: JAMA Health Forum. 2025 May 16;6(5):e252001. doi: 10.1001/jamahealthforum.2025.2001 (PMC12084836; doi:10.1001/jamahealthforum.2025.2001)
Supplement: Supplement 2. — Data Sharing Statement [file jamahealthforum-e252001-s002.pdf]

Data Sharing Statement

Ganguli. Gender Differences in Primary Care Physician Earnings and Outcomes Under Medicare Advantage Value-Based Payment. *JAMA Health Forum*. Published May 16, 2025. doi:10.1001/jamahealthforum.2025.2001

Data
Data available: No
